# The Emergency Resection of a Mobile Left Ventricular Thrombus in Dilated Cardiomyopathy: A Case of Multiorgan Embolism and Postoperative Ventricular Tachycardia

**DOI:** 10.7759/cureus.89756

**Published:** 2025-08-10

**Authors:** Soichiro Ota, Yuki Hayashi, Keita Kamata, Naoki Eguchi, Masashi Tanaka

**Affiliations:** 1 Cardiovascular Surgery, Nihon University School of Medicine, Tokyo, JPN

**Keywords:** dilated cardiomyopathy, heart failure, left ventricular thrombus, multiorgan infarction, surgical thrombectomy, systemic embolism, ventricular tachycardia

## Abstract

Dilated cardiomyopathy (DCM) is characterized by marked left ventricular (LV) dilation and systolic dysfunction, which predispose patients with DCM to LV thrombus (LVT) formation and potentially fatal multiorgan embolism. We report the case of a 52-year-old male with severe DCM (ejection fraction (EF), 14%; LV diameter, 89 mm) who developed a large, mobile LVT (40 × 30 mm) that caused embolic events in the lungs, kidneys, spleen, superior mesenteric artery, and posterior cerebral cortex. Since anticoagulation therapy was deemed insufficient to control the embolic source, emergency surgical thrombectomy was performed via an apical LV incision. Although the patient's condition initially stabilized, he developed ventricular tachycardia and torsades de pointes on postoperative day (POD) nine, which led to cardiac arrest. He was successfully resuscitated but required intensive antiarrhythmic therapy and circulatory support. The patient was discharged on POD 41 with an implantable cardioverter-defibrillator. This report illustrates that surgical removal of a large, mobile LVT is effective in controlling embolic events; however, an LV incision may induce postoperative life-threatening ventricular arrhythmias due to myocardial scarring and electrical heterogeneity. Rigorous perioperative monitoring and comprehensive arrhythmia management are essential when surgical thrombectomy is considered for LVT related to DCM.

## Introduction

Dilated cardiomyopathy (DCM) is a nonischemic cardiomyopathy defined by progressive left ventricular (LV) dilation and impaired systolic function and is recognized as one of the leading causes of heart failure. Its prevalence in the general population is estimated at approximately one in 250 individuals in Western countries [1]. Structural remodeling and hemodynamic changes in DCM promote intracavitary blood stasis and endocardial damage, predisposing patients with DCM to LV thrombus (LVT). The incidence of LVT in nonischemic cardiomyopathy ranges from 10% to 20% and may be higher in patients with advanced LV dysfunction [2,3].

While anticoagulant therapy is the first-line treatment for LVT, surgical thrombectomy is considered in cases where the thrombus is large, mobile, or associated with systemic embolism and when anticoagulation fails to prevent further embolization [4,5]. However, few reports have detailed the postoperative course of LVT resection in DCM patients, particularly concerning arrhythmic complications. We describe a case of emergency surgical thrombectomy in a patient with advanced DCM and multiple embolic complications who subsequently developed life-threatening postoperative ventricular arrhythmias. This report highlights both the therapeutic potential and risks associated with surgical management of LVT in DCM.

## Case presentation

A 52-year-old male (body surface area (BSA): 2.00 m²) with a medical history of asthma, coronavirus disease 2019 pneumonia, and sleep apnea syndrome presented with worsening orthopnea. He had previously been evaluated at our cardiology department for heart failure and suspected DCM; however, follow-up had been interrupted. One month before admission, the patient had experienced progressive dyspnea. Despite outpatient management, his symptoms had persisted, and he was admitted with acute decompensated heart failure and pneumonia. On hospital day two, transthoracic echocardiography revealed an ejection fraction (EF) of 17%, and dobutamine administration was initiated. His hemodynamic status improved, and inotropic support was tapered by day seven.

**Figure 1:**
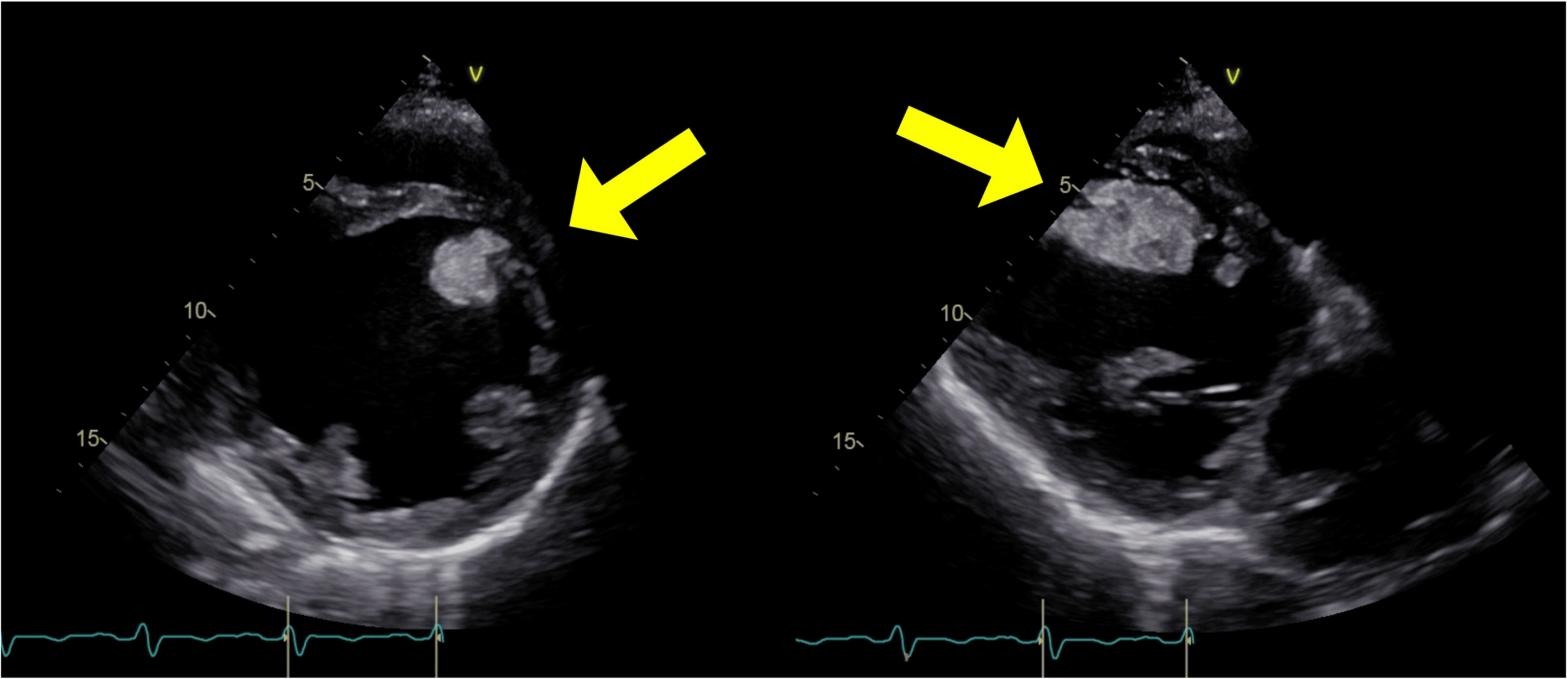
Transthoracic echocardiography The left panel shows a short-axis view of the left ventricle, and the right panel displays a long-axis view. A large, mobile thrombus is clearly visualized within the left ventricular cavity

**Figure 2:**
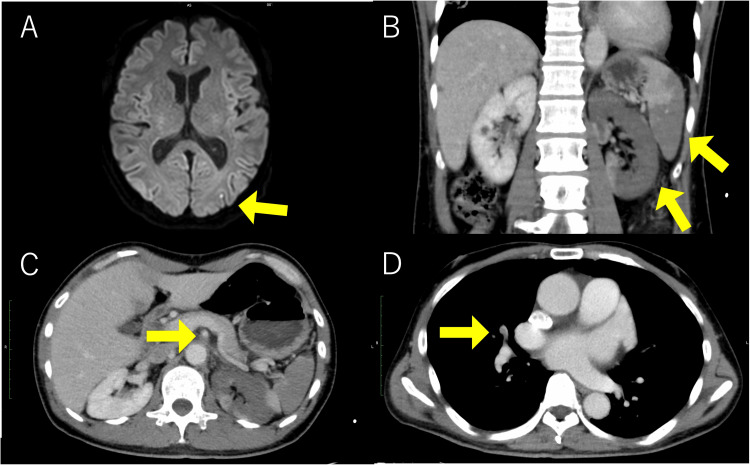
Contrast-enhanced CT and MRI findings (A) Diffusion-weighted MRI showing high signal intensity consistent with acute cerebral infarction in the left occipital lobe. (B) Contrast-enhanced CT demonstrating infarctions in the kidneys and spleen. (C) A superior mesenteric artery (SMA) thrombus is identified. (D) A contrast defect is observed in the right pulmonary artery, indicative of pulmonary embolism CT: computed tomography; MRI: magnetic resonance imaging

However, on day eight, the patient developed sudden-onset left lower abdominal and flank pain. Initial CT findings were unremarkable, and ureteral colic was suspected. By day nine of hospitalization, his symptoms persisted, and laboratory testing revealed an elevated D-dimer level and worsening renal function. Repeat echocardiography revealed a large, protruding, and mobile LVT measuring 40 × 30 mm, extending into the ventricular cavity (Figure 1). Contrast-enhanced CT revealed segmental embolic occlusions in the pulmonary artery, as well as emboli in the spleen, kidneys, and superior mesenteric artery. Brain MRI revealed an acute infarction in the left occipital cortex (Figure 2). Given the thrombus size, its mobility, and the presence of ongoing embolic events, an emergency surgical thrombectomy was undertaken on the day of diagnosis.

On presentation to the surgical unit, his vital signs were stable. Laboratory testing revealed elevated inflammatory markers, renal dysfunction, and increased D-dimer levels (Table 1). Chest radiography revealed a cardiothoracic ratio of 62.9%, without pleural effusion. Transthoracic echocardiography showed an EF of 14.1% and marked LV dilation (89.4 mm), with a large, mobile thrombus visualized on the anterior wall.

**Table 1:** Laboratory investigations ALT: alanine aminotransferase; AST: aspartate aminotransferase; CRP: C-reactive protein

Parameters	On the day of surgery	Reference values
Total leukocytes (x10/uL)	13.7	4.0-10.0
Hematocrit (%)	51.9	40-50
Hemoglobin (gm/dL)	17.2	13-17
Platelet (x10^3^/uL)	304	150-410
Serum urea (mmol/L)	24.7	2.5-7.8
Serum creatinine (umol/L)	1.66	62-106
Serum potassium K (mmol/L)	4.1	3.5-5.3
Serum sodium (mmol/L)	136	133-146
ALT (IU/L)	25	0-41
AST (IU/L)	20	0-41
CRP (mg/L)	1.76	0-5
D-dimer (mg/L FEU)	12.6	<0.5

**Figure 3:**
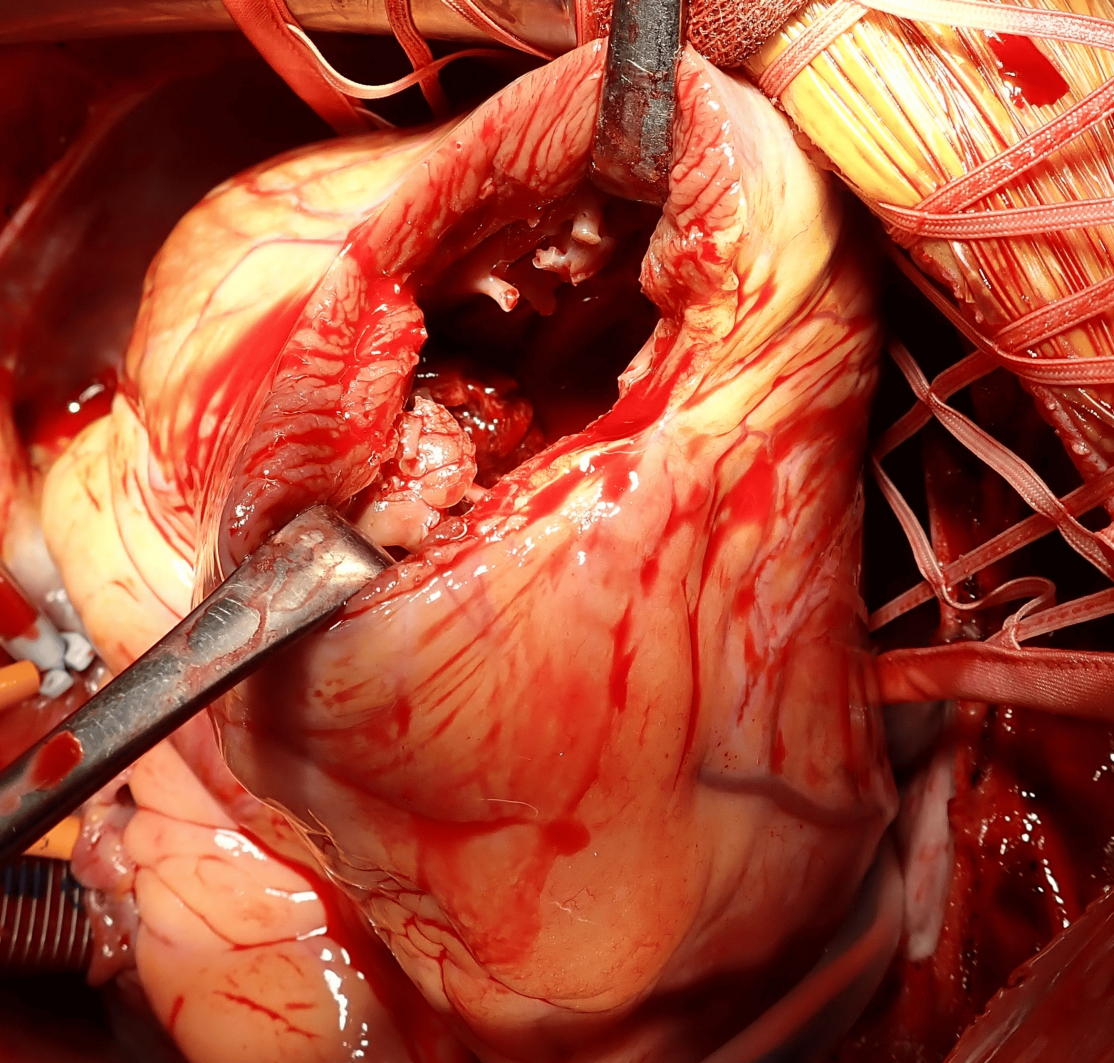
Intraoperative view of left ventricular thrombus Intraoperative photograph showing the incised left ventricle with the thrombus visualized in situ, consistent with preoperative echocardiographic findings

**Figure 4:**
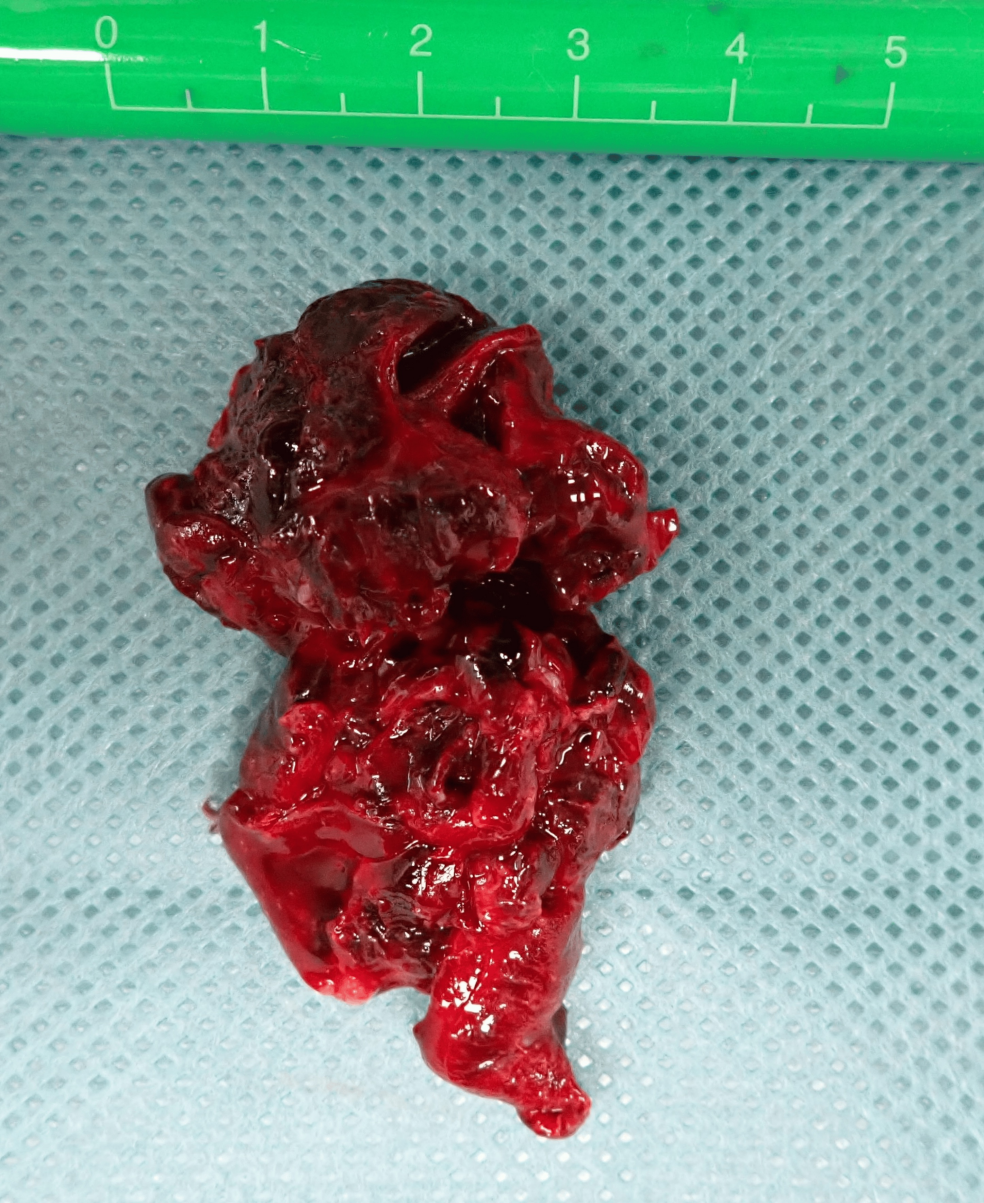
Resected left ventricular thrombus Gross specimen of the resected thrombus, measuring approximately 40 × 30 mm

Under general anesthesia and via a median sternotomy, cardiopulmonary bypass was established with ascending aortic cannulation and right atrial drainage. After achieving cardiac arrest under ventricular fibrillation, an apical LV incision approximately 7 cm in length was made parallel to the left anterior descending artery (Figure 3). This apical approach was chosen because the thrombus was located near the apex, and it provided the most direct access and optimal visualization. A large brown thrombus was observed and removed under direct visualization (Figure 4). The papillary muscle cavity was dilated and carefully debrided, and the ventricular incision was reinforced using felt pledgets and double-layer suturing. Transesophageal echocardiography was employed intraoperatively to assist with surgical guidance. No mechanical support was required, and the patient was weaned from cardiopulmonary bypass uneventfully. The surgical duration was two hours and 53 minutes, the bypass time was 85 minutes, and the aortic cross-clamp time was 43 minutes. No blood transfusions were performed.

The patient was extubated on postoperative day (POD) one. On POD four, premature ventricular contractions were observed without hemodynamic compromise. The patient was transferred to the ICU on POD seven. On the night of POD eight, the patient developed atrial tachycardia (heart rate: 140 bpm) and was initially treated with digoxin, given its rapid onset of action and minimal effect on blood pressure despite the patient's severely reduced cardiac function. However, as the heart rate remained uncontrolled after two hours, amiodarone was subsequently initiated at a maintenance dose. On POD nine, the patient experienced sudden cardiac arrest due to ventricular tachycardia (VT) and torsades de pointes, requiring defibrillation and resuscitation for 17 minutes. Emergency coronary angiography revealed no obstructive lesions. He was intubated, and intra-aortic balloon pumping was initiated. Continuous renal replacement therapy was started on POD 12 and discontinued on POD 15. The patient was extubated on POD 17 and discharged from the CCU on POD 20. CRT-D was implanted on POD 32, and the patient was discharged on POD 41.

## Discussion

DCM leads to structural and electrical remodeling, which predisposes patients with DCM to thrombus formation and arrhythmias. In our patient, a severely reduced EF (14%) and markedly enlarged LV cavity (LV dilation: 89 mm) were significant risk factors for thrombus development. Hemodynamic stasis, endocardial injury, and hypercoagulability in the context of acute decompensation likely contributed to thrombus formation. Although anticoagulation is the standard of care for LVT, surgical removal may be necessary when embolic events occur despite therapy or when thrombus characteristics suggest imminent risk [4,5]. In our case, the mobile nature and large size of the thrombus, along with documented multiorgan embolism, justified surgical intervention. The left apical approach enabled direct visualization and safe removal of the thrombus.

However, the risk of postoperative arrhythmia is a critical concern in such cases. An LV incision may induce electrical heterogeneity and scar formation, providing a substrate for reentrant arrhythmias [6,7]. Myocardial injury from the incision, along with ischemia caused by suturing and aortic cross-clamping, may also contribute. These factors may explain the recurrent and life-threatening VT observed postoperatively in our patient. This risk may be particularly elevated in patients with DCM, where pre-existing electrical instability due to extensive myocardial fibrosis and ventricular dilation already provides an arrhythmogenic substrate. Although direct evidence is limited, it is conceivable that apical ventriculotomy in such structurally remodeled hearts could further disrupt conduction pathways-especially in areas of wall thinning or mid-myocardial scarring-and thereby increase the susceptibility to postoperative arrhythmias. Furthermore, this case underscores the importance of long-term follow-up to monitor for recurrent thromboembolism, late-onset arrhythmias, and overall cardiac function recovery.

To the best of our knowledge, only three prior case reports have described surgical thrombectomy for LVT in patients with DCM [8-10]. Naruse et al. reported two cases of cerebral embolism, whereas Chu and Mukai described favorable outcomes following resection of prominent mobile thrombi. None of the patients experienced fatal postoperative arrhythmias. By contrast, our patient developed sustained VT and torsades de pointes requiring resuscitation and subsequent implantable cardioverter-defibrillator implantation, underscoring the importance of perioperative arrhythmia surveillance and prophylaxis. This report provides novel clinical insights into both the therapeutic value and potential risks of LVT resection in DCM. It highlights the need for comprehensive management strategies that address not only embolic risk but also the potential for postoperative arrhythmias.

## Conclusions

LVT in patients with severe DCM is a life-threatening condition associated with systemic embolism. Although surgical resection is an effective strategy for large, mobile thrombi, it carries the risk of inducing ventricular arrhythmias owing to postoperative myocardial injury and scar formation. Our case underscores the necessity of rigorous perioperative arrhythmia management, including consideration of implantable cardioverter-defibrillator implantation. This report contributes valuable evidence to the limited literature on surgical thrombectomy for DCM and emphasizes the need for vigilance regarding arrhythmic complications.
